# Supplementary material for: CHEX-seq detects single-cell genomic single-stranded DNA with catalytical potential
Source: Nat Commun. 2023 Nov 14;14:7346. doi: 10.1038/s41467-023-43158-6 (PMC10645931; doi:10.1038/s41467-023-43158-6)
Supplement: Supplementary file 1 — Supplementary Information [file 41467_2023_43158_MOESM1_ESM.pdf]

Supplementary Information for manuscript

CHEX-seq Detects Single-Cell Genomic Single-Stranded DNA With Catalytical Potential

Table of Contents

Supplementary Figures

Supplementary Figure 1. Chemical synthesis workflow of the CHEX-seq probes.

Supplementary Figure 2. Illustration of the single-stranded open chromatin model and the CHEX-seq protocol.

Supplementary Figure 3. Definition of the CHEX-seq barcode/primer quality classes and subclasses under A, B and C.

Supplementary Figure 4. Quality assessment of CHEX-seq priming counts, TSS coverage, genomic distribution, and degree of enrichment in ssDNA or transcripts of K562 non-control and control samples.

Supplementary Figure 5. Comparison between CHEX-seq and ATAC-seq in TSS coverage profiles using reads extended to the same size (2kb) for CHEX-seq and ATAC-seq.

Supplementary Figure 6. CHEX-epigenome association analysis.

Supplementary Figure 7. FISH validation of single-stranded, intergenic loci in K562.

Supplementary Figure 8. CHEX-seq coverage profiles in gene-body, 5' UTR, TSS and CpG Island flanking regions in mouse neuron and interneuron tissue section samples.

Supplementary Figure 9. Correlation between CHEX-seq priming distance to the TSS and RNA-seq gene expression.

Supplementary Figure 10. Overlap between CHEX-seq and transcriptome from the corresponding cell type in various sub-genic regions.

Supplementary Figure 11. Mitochondrial priming patterns in human and mouse.

Supplementary Figure 12. Single-base strand specific priming counts in human and mouse mitochondrial genome.

Supplementary Tables

Supplementary Table 1. List of the oligo sequences of CHEX-seq probes, barcodes, and primers.

Supplementary Table 2. List of the oligo sequences for gDNAzyme analysis.

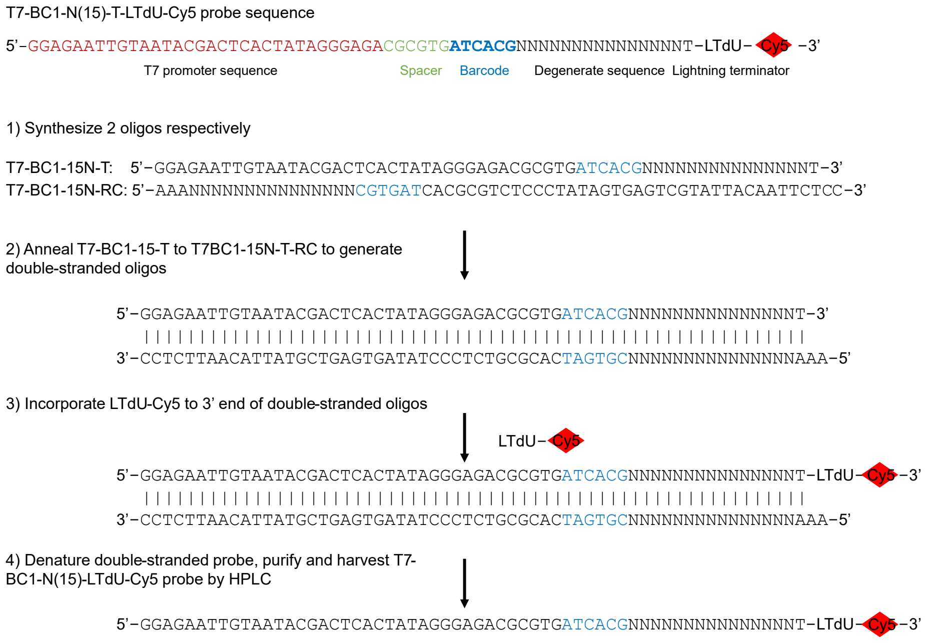

Supplementary Figure 1. Chemical synthesis workflow of the CHEX-seq probes (BC1 for example. For the complete barcode/primer list see **Supplementary Table 1**).

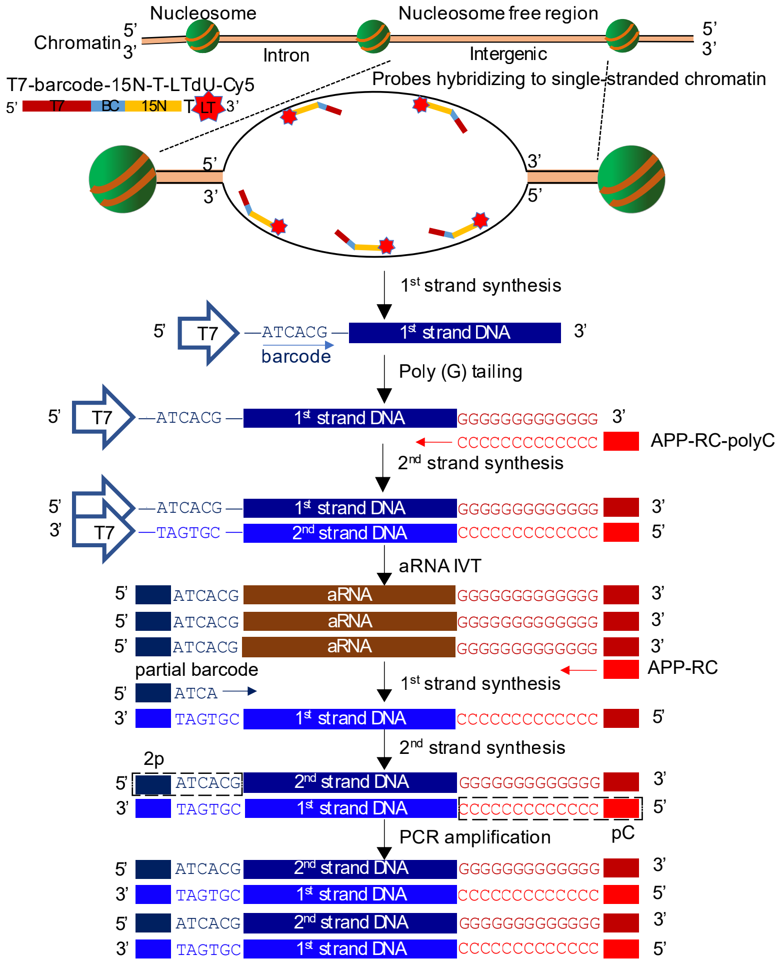

Supplementary Figure 2. Illustration of the single-stranded open chromatin model and the CHEX-seq protocol. Dashed-line boxes denote the two sequences that define the CHEX-seq barcode/primer quality. The bold font denotes the barcode/primer identifier (BC1 for example. For the complete barcode/primer list see **Supplementary Table 1**).

Class	A: Insert bracketed by primers both present & proper		B: Only barcode primer (bP) present & proper		C: Only non-barcode primer (nP) present & proper		D: None of primers present or proper
	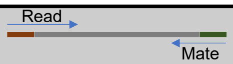 Read / Mate		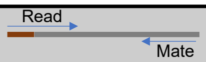 Read / Mate		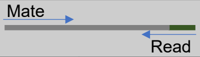 Mate / Read		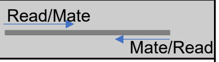 Read/Mate / Mate/Read
Subclass	A1 (long fragment)	A2 (short fragment)	B1 (long fragment)	B2 (short fragment)	C1 (long fragment)	C2 (short fragment)	--
SE	--		bP present & proper*		nP present & proper*		Remaining reads
			nP(rc) not present	nP(rc) present & proper	bP(rc) not present	bP(rc) present & proper	
PE	bP present & proper in one read* AND nP present & proper in the other		bP present & proper in one read* AND nP not present or proper in the other		nP present & proper in one read* AND bP not present or proper in the other		Remaining reads
	Primer reverse complements not present	nP(rc) present & proper in read containing bP OR bP(rc) present & proper in read containing nP	Primer reverse complements not present	nP(rc) present & proper in read containing bP	Primer reverse complements not present	bP(rc) present & proper in read containing nP	

bP = primer containing the barcode
nP = primer that does not contain the barcode
(rc) = reverse complement
* Criteria that if a read meets, it's called "Read", the other (if PE) called "Mate". For determining which read in a pair to be output if the insert belongs to a quality category.

Requirements for a "proper" primer: 6 <= presence length <= full length
For a "proper" primer(rc): 0 < presence length <= full length of the other primer

Supplementary Figure 3. Definition of the CHEX-seq barcode/primer quality classes and subclasses under A, B and C.

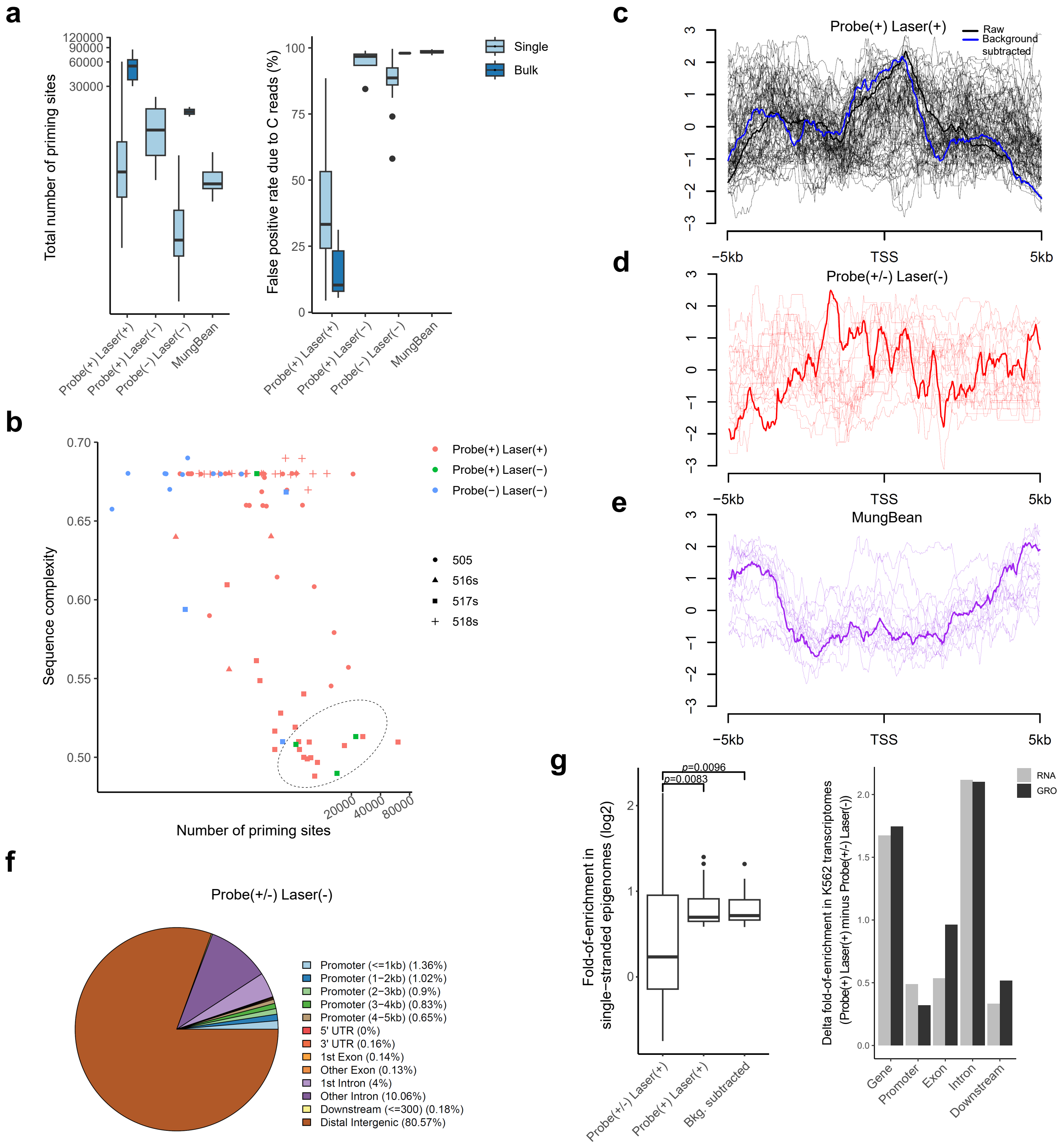

Supplementary Figure 4. Quality assessment of CHEX-seq priming counts, TSS coverage, genomic distribution, and degree of enrichment in ssDNA or transcripts of K562 non-control and control samples. (a) Left, total number of priming sites in non-control K562 and three types of controls. Right, fraction of C reads in non-control K562 and three types of controls; (b) Total number of priming sites (x-axis) and sequence complexity (y-axis) of non-control K562, Probe(+) Laser(-) and Probe(-) Laser(-) control samples. The shape of data points indicates the probe ID; (c-e) Priming coverage enrichment around TSS +/-5kb region in (c) non-control K562, (d) Probe(+/-) Laser(-) controls and (e) mung bean digested controls. Light thin lines indicate individual cells while dark thick lines indicate aggregated coverages pooling all samples for each group; (f) Genomic distribution of priming sites in Probe(+/-) Laser(-) controls; (g) Left, enrichment of priming sites in ENCODE ssDNA related epigenomes in non-control K562, including or excluding the background, or in Probe(+/-) Laser(-) controls (p-values from Wilcoxon's rank-sum test, two-sided). Right, enrichment of priming sites in K562 RNA-seq or GRO-seq transcriptomes in non-control K562, including or excluding the background, or in Probe(+/-) Laser(-) controls. For boxplots in (a) and (g), the bounds of the box represent the 1st and the 3rd quartile; the thick bar represents the median; the whiskers extend 1.5 times the IQR; the dots represent data points outside 1.5 times the IQR.

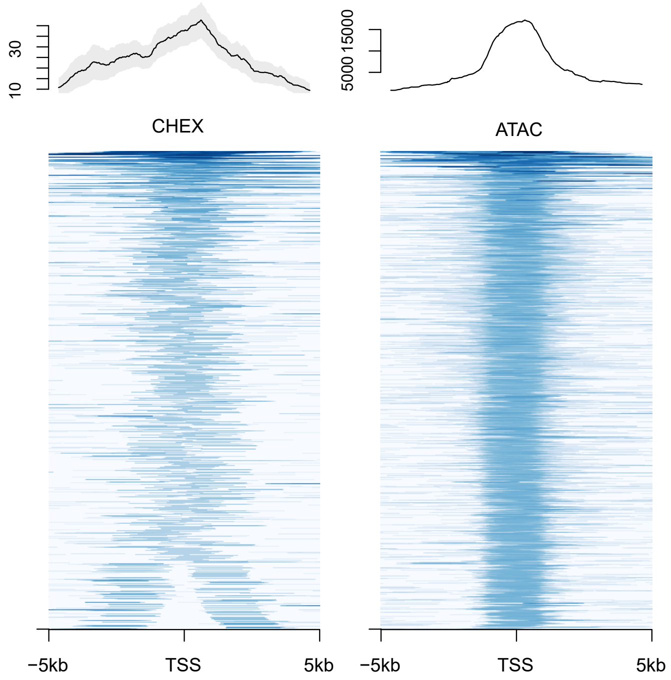

Supplementary Figure 5. Comparison between CHEX-seq and ATAC-seq in TSS coverage profiles using reads extended to the same size (2kb) for CHEX-seq and ATAC-seq. Both assays have mitochondrial and chrY reads removed. The coverage of top 1% (n=581) high-coverage genes across 500 bins within TSS ±5kb is shown in the heatmap (bottom), while gene-averaged coverage is shown as the curve (top); the shade over the curve presents the mean±SEM.

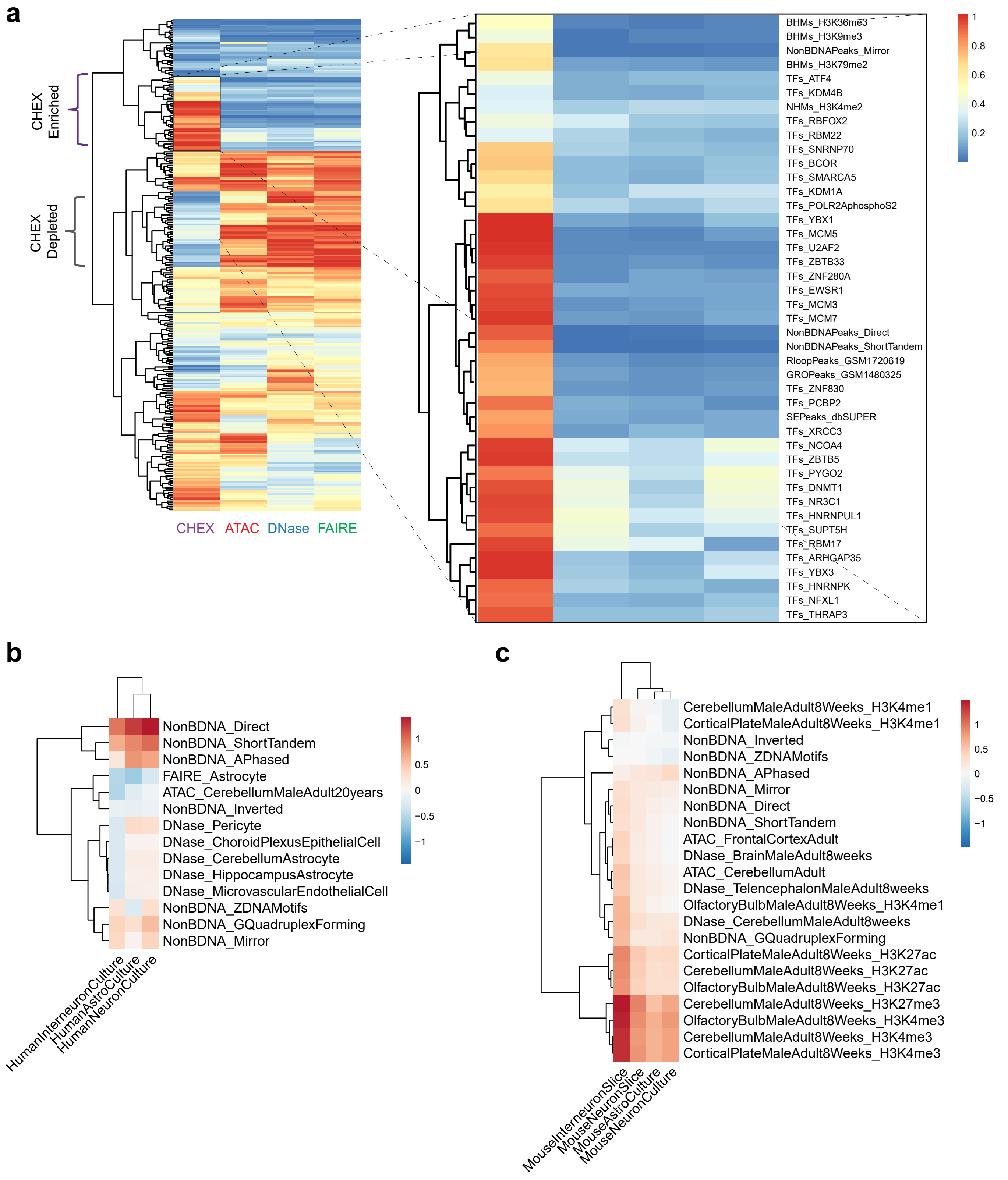

Supplementary Figure 6. CHEX-epigenome association analysis. (a) Hierarchical clustering of CHEX-, ATAC-, DNase- and FAIRE-seq by the similarity with the extended set of 284 K562 epigenomes. CHEX-seq reads have been filtered with more stringent criteria than Fig. 2c. Color indicates quantile normalized fold of enrichment per assay (0 means the lowest enrichment and 1 means the highest enrichment). NHM: Narrow Histone Marks. BHM: Broad Histone Marks; (b) CHEX-seq association with non-B-form DNA, ATAC-, DNase- and FAIRE-seq in human brain; color indicates the fold of enrichment in log2; (c) CHEX-seq association with non-B-form DNA, ATAC-, DNase-seq and ENCODE histone marks in mouse brain (8 weeks adult); color indicates the fold of enrichment in log2.

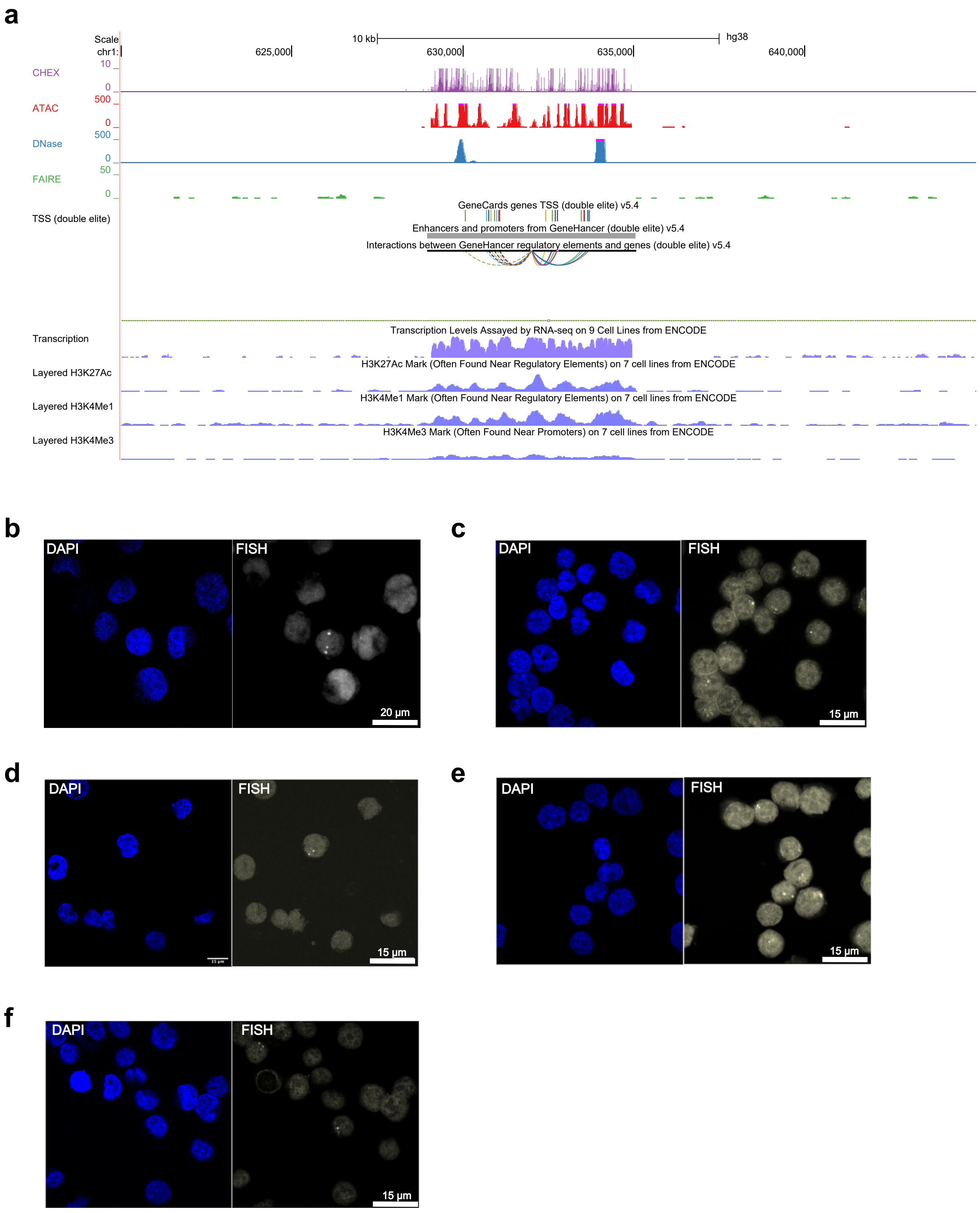

Supplementary Figure 7. FISH validation of single-stranded, intergenic loci in K562. (a) UCSC Genome Browser visualization of read coverage from CHEX-, ATAC-, DNase-, and FAIRE-seq; the lower panel shows GeneCards TSS, GeneHancer enhancers and promoters as well as chromatin interactions in between; (b) DAPI and FISH images of the K562 cells showing three annealing sites at the locus in (a), possibly due to the trisomy chromosomes in K562; (c-f) Additional FISH validation for CHEX-seq identified loci at (c) chr4:1466001-1468000, (d) chr8:116514001-116516000, (e) chr11:123002001-123004000, (f) chrX:55182001-55184000.

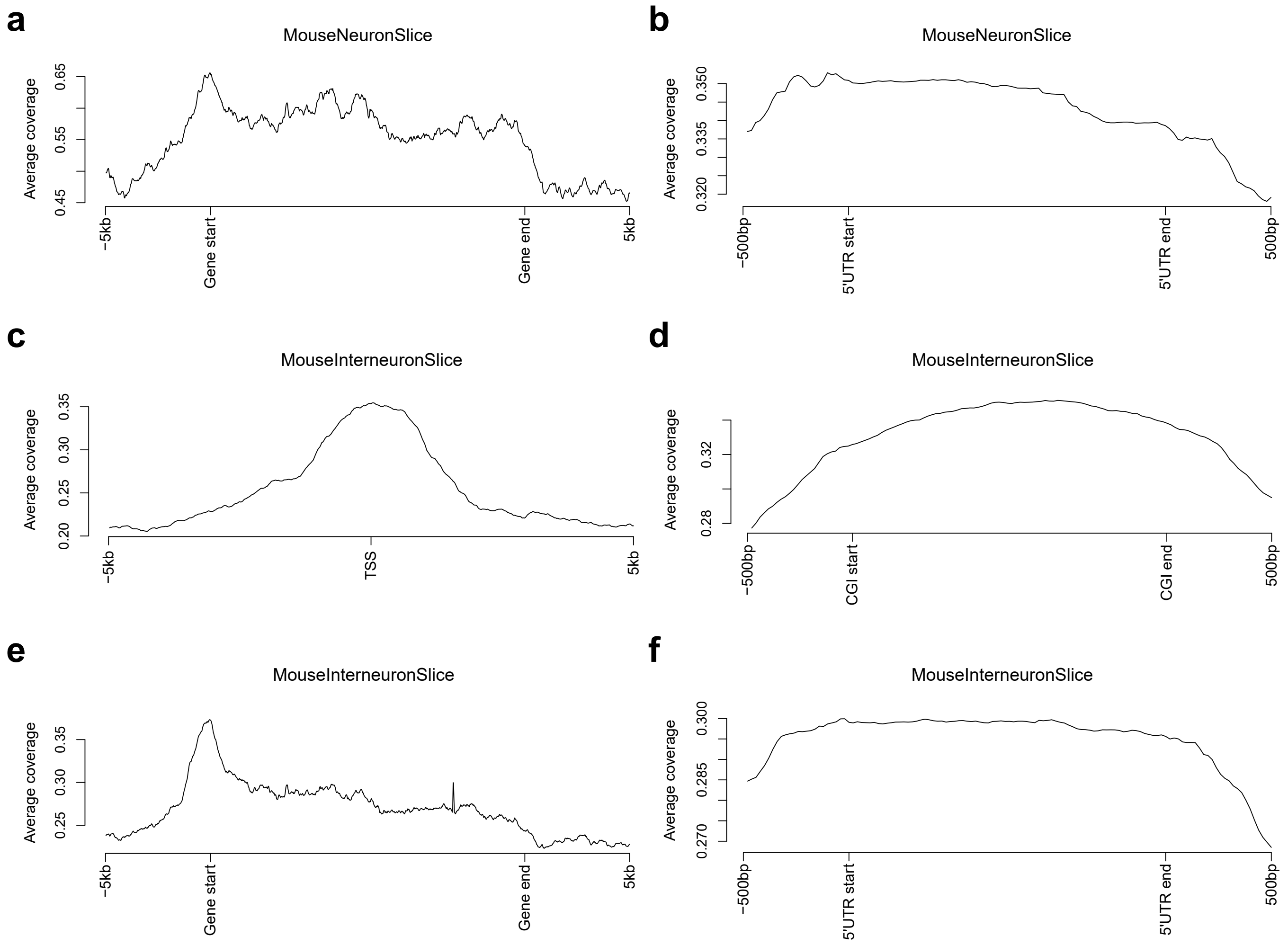

Supplementary Figure 8. CHEX-seq coverage profiles in gene-body, 5’ UTR, TSS and CpG Island flanking regions in mouse neuron and interneuron tissue section samples. (a) Gene-body ±5kb flanking in mouse neuron tissue section; (b) 5’ UTR ±500bp flanking in mouse neuron tissue section; (c) TSS ±5kb flanking in mouse interneuron tissue section; (d) CpG island ±500bp flanking in mouse interneuron tissue section; (e) Gene-body ±5kb flanking in mouse interneuron tissue section; (f) 5’ UTR ±500bp flanking in mouse interneuron tissue section.

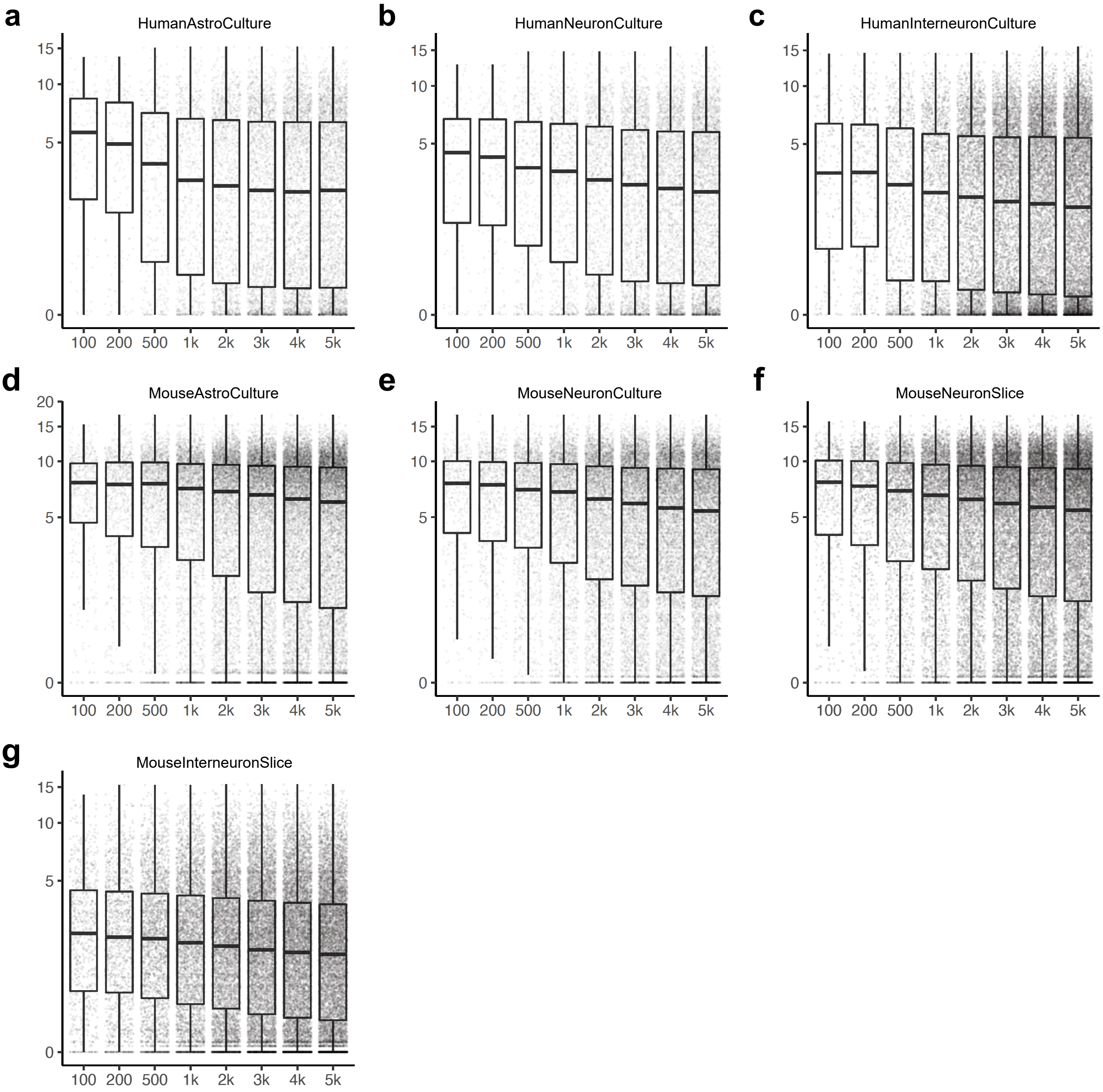

Supplementary Figure 9. Correlation between CHEX-seq priming distance to the TSS (x-axis) and RNA-seq gene expression (y-axis). (a) Human astrocyte culture; (b) Human neuron culture; (c) Human interneuron culture; (d) Mouse astrocyte culture; (e) Mouse neuron culture; (f) Mouse neuron tissue section; (g) Mouse interneuron tissue section. The bounds of the box represent the 1st and the 3rd quartile; the thick bar represents the median; the vertical line extends 1.5 times the IQR; the dots represent all data points including maxima and minima.

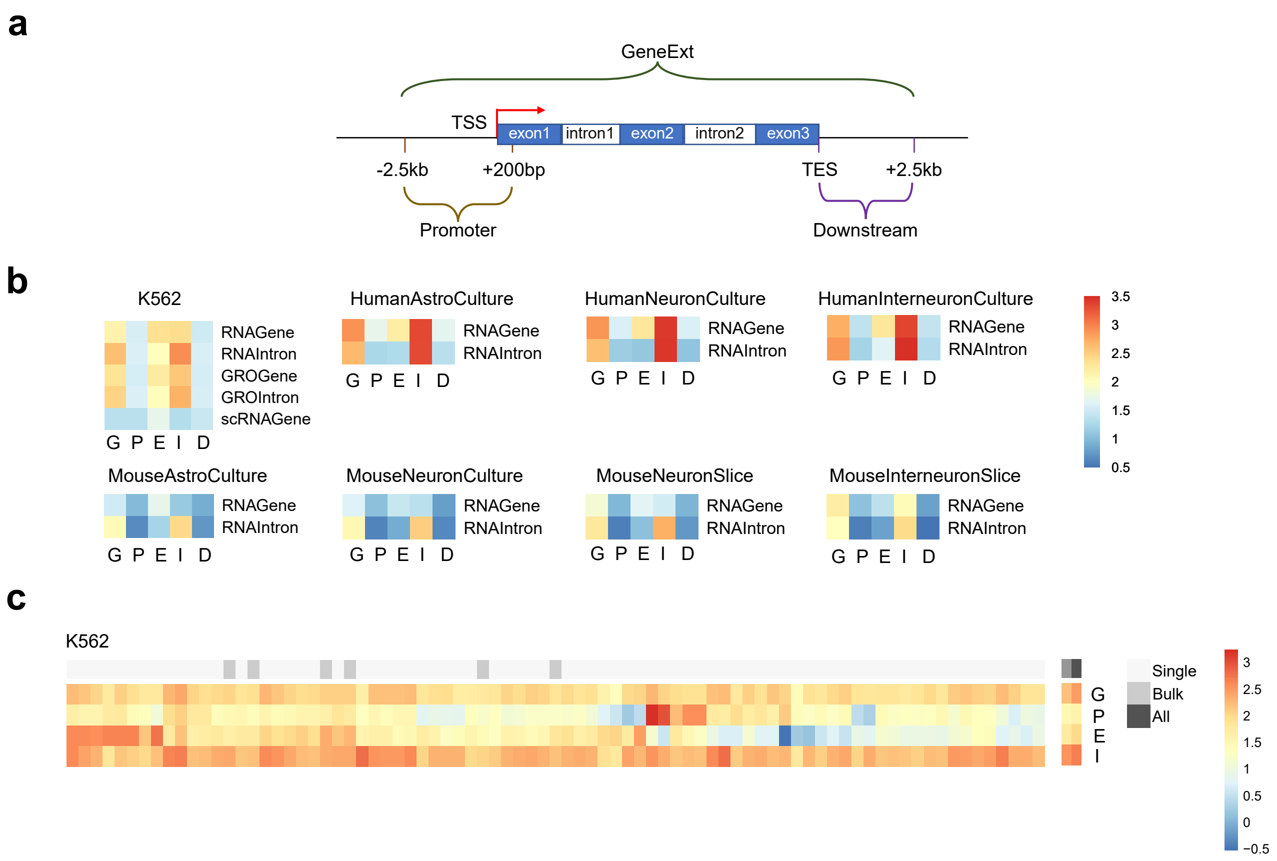

Supplementary Figure 10. Overlap between CHEX-seq and transcriptome from the corresponding cell type in various sub-genic regions. (a) Definition of the various sub-genic regions. TES: transcription end site; (b) Odds ratio (log2) for the overlap between CHEX-seq primed genes (cells aggregated, binarized at zero) and highly expressed genes (binarized at the median) of matched cell types (except for human interneuron where human neuron RNA-seq is used) in sub-genic regions ([G]eneExt, [P]romoter, [E]xon, [I]ntron, [D]ownstream); (c) Odds ratio (log2) for the overlap between CHEX-seq primed genes (binarized at zero) and highly expressed nascent RNAs (GRO-seq binarized at the median) in K562, showing single-cell, bulk and aggregated data.

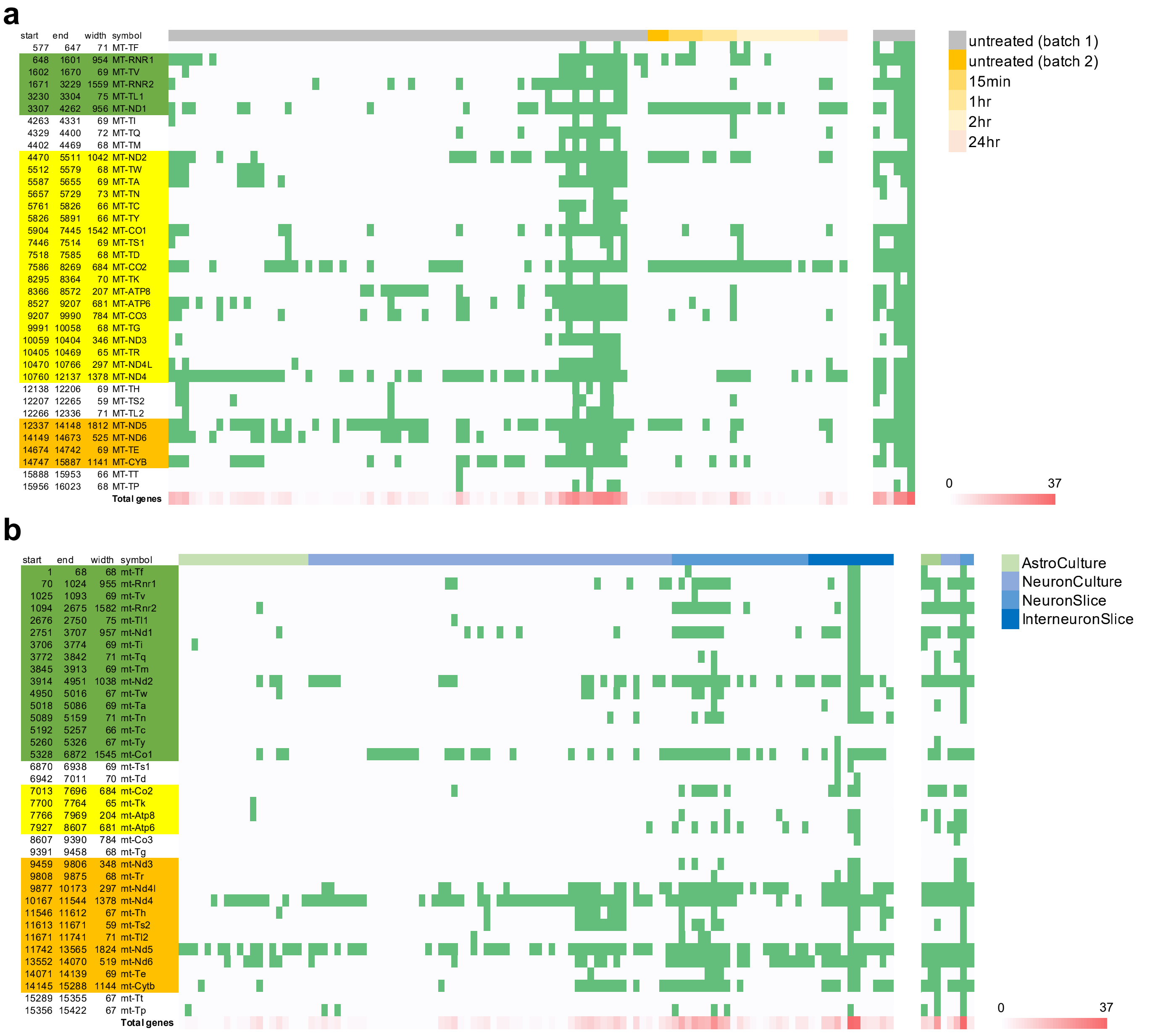

Supplementary Figure 11. Mitochondrial priming patterns in human and mouse. (a) Single-cell and bulk samples' priming status (binarized: green if primed) for 37 human mitochondrial encoded genes in K562 untreated and TPA treated samples; (b) Single-cell and population samples' priming status (binarized: green if primed) for 37 mouse mitochondrial encoded genes in mouse brain samples.

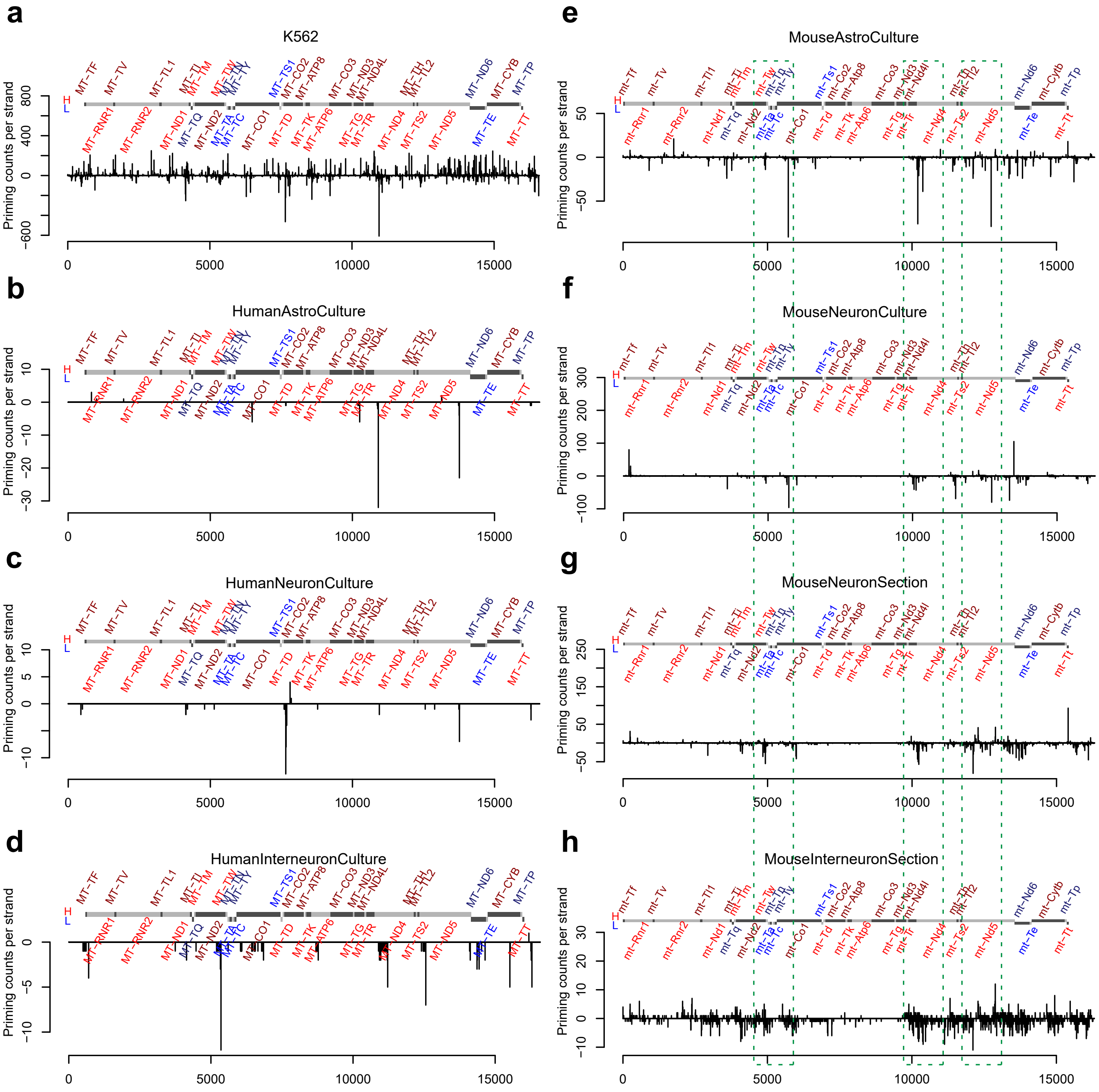

Supplementary Figure 12. Single-base strand specific priming counts in human and mouse mitochondrial genome. (a) K562; (b) Human astrocyte culture; (c) Human neurons in culture; (d) Human interneurons in culture; (e) Mouse astrocytes in culture; (f) Mouse neurons in culture; (g) Mouse neuron in brain section; (h) Mouse interneuron in brain section. X-axis: coordinate (bp) of the mitochondrial genome; y-axis: per-base priming counts in the plus (y > 0) and minus (y < 0) strand of the mitochondrial genome. Green dashed boxes highlight regions sharing strand-specific priming across mouse brain samples. The dark-light alternating font color corresponds to the shade of the gray boxes, to make adjacent genes more discernible.

ID	Name	Sequence
302	CHEX-App-RC-polyC	5′- GC GCC ATT GAC CAG GAT TTT CCC CCC CCC CCC CC -3′
303	CHEX-App-RC	5′- GC GCC ATT GAC CAG GAT TTT C -3′
505	CHEX-20bpBC1	5′- TAG GGA GAC GCG TG**A TCA CG** -3′
510	CHEX-15NT-2S-BC15	5′- GGA GAA TTG TAA TAC GAC TCA CTA TAG GGA GAC GCG TG**A TGT CA**acaat ga gga NNNNNNNNNNNNNNNT -3′
511	CHEX-15NT-2S-BC16	5′- GGA GAA TTG TAA TAC GAC TCA CTA TAG GGA GAC GCG TG**C CGT CC**acaat ga gga NNNNNNNNNNNNNNNT -3′
512	CHEX-15NT-2S-BC17	5′- GGA GAA TTG TAA TAC GAC TCA CTA TAG GGA GAC GCG TG**G TAG AG**acaat ga gga NNNNNNNNNNNNNNNT -3′
513	CHEX-15NT-2S-BC19	5′- GGA GAA TTG TAA TAC GAC TCA CTA TAG GGA GAC GCG TG**G TGA AA**acaat ga gga NNNNNNNNNNNNNNNT -3′
514	CHEX-15NT-2S-BC20	5′- GGA GAA TTG TAA TAC GAC TCA CTA TAG GGA GAC GCG TG**G TGG CC**acaat ga gga NNNNNNNNNNNNNNNT -3′
515	CHEX-15NT-2S-BC21	5′- GGA GAA TTG TAA TAC GAC TCA CTA TAG GGA GAC GCG TG**G TTT CG**acaat ga gga NNNNNNNNNNNNNNNT -3′
516	CHEX-15NT-2S-BC22	5′- GGA GAA TTG TAA TAC GAC TCA CTA TAG GGA GAC GCG TG**C GTA CG**acaat ga gga NNNNNNNNNNNNNNNT -3′
517	CHEX-15NT-2S-BC23	5′- GGA GAA TTG TAA TAC GAC TCA CTA TAG GGA GAC GCG TG**G AGT GG**acaat ga gga NNNNNNNNNNNNNNNT -3′
518	CHEX-15NT-2S-BC24	5′- GGA GAA TTG TAA TAC GAC TCA CTA TAG GGA GAC GCG TG**G GTA GC**acaat ga gga NNNNNNNNNNNNNNNT -3′
505b	CHEX-18bpPBC1 (short BC1)	5′- TAG GGA GAC GCG TG**A TCA** -3′
505c	CHEX-17bpPBC1 (short BC1)	5′- TAG GGA GAC GCG TG**A TC** -3′
505d	CHEX-16bpPBC1 (short BC1)	5′- TAG GGA GAC GCG TG**A T** -3′
507b	CHEX-18bpPBC14	5′- TAG GGA GAC GCG TG**A GTT** -3′
510b	CHEX-18bpPBC15	5′- TAG GGA GAC GCG TG**A TGT** -3′
511b	CHEX-18bpPBC16	5′- TAG GGA GAC GCG TG**C CGT** -3′
512b	CHEX-18bpPBC17	5′- TAG GGA GAC GCG TG**G TAG** -3′
513b	CHEX-18bpPBC19	5′- TAG GGA GAC GCG TG**G TGA** -3′
514b	CHEX-18bpPBC20	5′- TAG GGA GAC GCG TG**G TGG** -3′
515b	CHEX-18bpPBC21	5′- TAG GGA GAC GCG TG**G TTT** -3′
516b	CHEX-18bpPBC22	5′- TAG GGA GAC GCG TG**C GTA** -3′
517b	CHEX-18bpPBC23	5′- TAG GGA GAC GCG TG**G AGT** -3′
518b	CHEX-18bpPBC24	5′- TAG GGA GAC GCG TG**G GTA** -3′
529n	CHEX-18bp-NBC	5′- A GAC GCa gaa gag caG TG -3′

Supplementary Table 1. List of the oligo sequences of CHEX-seq probes, barcodes, and primers. The blue bold font denotes the barcode (full or 3’ end-clipped partial version); the red font denotes the T7 promoter in CHEX-seq probes; The green uppercase denotes the first spacer, and the green lowercase denotes the second spacer in dual-spacer primers (i.e., 510-518).

	Primer name	Sequence
DNAzyme	TATDN2P1long	CAAAGAGCAGTCTATCTAGGCTGGTGTTTTCCACAAAATAGGGTGGGTGGG TGGGTTTTCTTTCACTCCCTTCACCATCTATCCTAATGTT
	TATDN2P1short	GTTTTCCACAAAATAGGGTGGGTGGGTGGGTTTTCTTTCACTCCCTTCA
	opTATDN2P1anneal-short-loop	AACATTAGGATAGATGGTGAAAAACACCAGCCTAGATAGACTGCTCTTTG
	opTATDN2P1anneal-long-loop	AACATTAGGATAGACTGCTCTTTG
	Bmpr1a	TAATAATGGATTGGGTGGGTGGGTGGGTACAAGGATGGAGATGA
	RPL7AP61	GTTTGATGGAAAAATGGGTGGGTGGGTGGGTAGATGAATGGATAGAT
FRET	Bmpr1aFishFRETProbes-3'	ccaatccattaaaattactct/3ATTO550N/
	Bmpr1aFishFRETProbes-5'	/5ATTO590N/cccttttagacctaaaaggtg

Supplementary Table 2. List of the oligo sequences for gDNAzyme analysis. The first set contains sequences used to test genomic DNA sequences for DNAzyme activity. The second set of sequences are the FISH-FRET probes for Bmpr1a.
