# Supplementary material for: CHEX-seq detects single-cell genomic single-stranded DNA with catalytical potential
Source: Nat Commun. 2023 Nov 14;14:7346. doi: 10.1038/s41467-023-43158-6 (PMC10645931; doi:10.1038/s41467-023-43158-6)
Supplement: Supplementary file 3 — Description of Additional Supplementary Files [file 41467_2023_43158_MOESM3_ESM.pdf]

Description of Additional Supplementary Files

File Name: Supplementary Data 1
Description: Sample metadata including the probe, primers, light activation, mung bean/TPA treatment, cell type information and harvest method for each sample.

File Name: Supplementary Data 2
Description: Number of CHEX-seq reads (pre-alignment) in each barcode-primer quality class (A, B, C, D) and subclass (A1, A2, B1, B2, C1, C2).

File Name: Supplementary Data 3
Description: Number of CHEX-seq aligned reads in each barcode-primer quality class (A, B, C, D, A merged with B [AB]) and for each alignment stringency level (mapped length $\geq$ 30, $\geq$ 20, $\geq$ 10, $\geq$ 20 but promiscuous regions masked [strict]). This table was used for determining the quality threshold.

File Name: Supplementary Data 4
Description: CHEX-epigenome genomic association fold-of-enrichment (odds ratio in log2) for K562 (A), human brain (B) and mouse brain (C) samples. The human and mouse epigenomes have their file accession number listed in (D) per ENCODE data reference policy.

File Name: Supplementary Data 5
Description: List of CHEX-seq predicted ssDNA loci selected for intergenic FISH validation.

File Name: Supplementary Data 6
Description: Porphyrin metalation DNAzymes' mimicking loci found as ssDNA in human and mouse. (A) List of the DNAzymes downloaded from the database DNAmoreDB; (B) Human genomic sites bearing homology (column O-U) to porphyrin metalation DNAzymes and the CHEX-seq priming counts inside (column AE); (C) Mouse genomic sites bearing homology (column O-U) to porphyrin metalation DNAzymes and the CHEX-seq priming counts inside (column AE).
