# Supplementary material for: CHEX-seq detects single-cell genomic single-stranded DNA with catalytical potential
Source: Nat Commun. 2023 Nov 14;14:7346. doi: 10.1038/s41467-023-43158-6 (PMC10645931; doi:10.1038/s41467-023-43158-6)
Supplement: Supplementary file 10 — Reporting Summary [file 41467_2023_43158_MOESM10_ESM.pdf]

Corresponding author(s): James Eberwine

Last updated by author(s): 2023 06 08

Reporting Summary

Nature Portfolio wishes to improve the reproducibility of the work that we publish. This form provides structure for consistency and transparency in reporting. For further information on Nature Portfolio policies, see our Editorial Policies and the Editorial Policy Checklist.

Statistics

For all statistical analyses, confirm that the following items are present in the figure legend, table legend, main text, or Methods section.

n/a	Confirmed	
☐	☒	The exact sample size (*n*) for each experimental group/condition, given as a discrete number and unit of measurement
☐	☒	A statement on whether measurements were taken from distinct samples or whether the same sample was measured repeatedly
☐	☒	The statistical test(s) used AND whether they are one- or two-sided<br>*Only common tests should be described solely by name; describe more complex techniques in the Methods section.*
☐	☒	A description of all covariates tested
☐	☒	A description of any assumptions or corrections, such as tests of normality and adjustment for multiple comparisons
☐	☒	A full description of the statistical parameters including central tendency (e.g. means) or other basic estimates (e.g. regression coefficient) AND variation (e.g. standard deviation) or associated estimates of uncertainty (e.g. confidence intervals)
☐	☒	For null hypothesis testing, the test statistic (e.g. *F*, *t*, *r*) with confidence intervals, effect sizes, degrees of freedom and *P* value noted<br>*Give P values as exact values whenever suitable.*
☒	☐	For Bayesian analysis, information on the choice of priors and Markov chain Monte Carlo settings
☐	☒	For hierarchical and complex designs, identification of the appropriate level for tests and full reporting of outcomes
☒	☐	Estimates of effect sizes (e.g. Cohen's *d*, Pearson's *r*), indicating how they were calculated

Our web collection on statistics for biologists contains articles on many of the points above.

Software and code

Policy information about availability of computer code

Data collection	High-throughput sequencing was done on Illumina NextSeq 500. UV-vis absorption spectra were recorded on a Lambda 365 UV-Vis spectrophotometer. Cells were screened for FRET detection using a Zeiss 880 confocal microscope.
Data analysis	Raw reads were demultiplexed by bcl2fastq v2.17, quality checked by FastQC v0.11.2 and BLAST v2.2.30, aligned by STAR v2.4.0h1. Afterwards, a custom pipeline (https://github.com/kimpenn/chex-seq) was used to assign barcode/primer quality, filter out low mapping-quality or contaminant reads, and output single-stranded open-chromatin sites in BED format. Data analysis scripts are public at https://github.com/kimpenn/chex-analysis. Also DOI for the CHEX-seq pipeline: 10.5281/zenodo.8399820 and DOI for the CHEX-seq data analysis: 10.5281/zenodo.8399827.

For manuscripts utilizing custom algorithms or software that are central to the research but not yet described in published literature, software must be made available to editors and reviewers. We strongly encourage code deposition in a community repository (e.g. GitHub). See the Nature Portfolio guidelines for submitting code & software for further information.

Data

Policy information about availability of data

All manuscripts must include a data availability statement. This statement should provide the following information, where applicable:

- Accession codes, unique identifiers, or web links for publicly available datasets
- A description of any restrictions on data availability
- For clinical datasets or third party data, please ensure that the statement adheres to our policy

Data has been deposited in GEO with the following accession number. GSE232216 (https://www.ncbi.nlm.nih.gov/geo/query/acc.cgi?&acc=GSE232216) Data will be made publicly available upon publishing of the paper. Also the CHEX-seq Pipeline is https://zenodo.org/record/8399820 and CHEX-seq Data Analysis is https://zenodo.org/record/8399827.   The raw CHEX-seq data (in FASTQ format) and the processed CHEX-seq data generated in this study, including the per-sample priming sites (in BED format) and the gene-by-sample priming count matrices, have been deposited in the GEO database under accession code GSE231719 [https://www.ncbi.nlm.nih.gov/geo/query/acc.cgi?acc=GSE231719]. The mouse brain single-cell RNA-seq data from primary culture and from acute slice have been deposited in the GEO database under accession code GSE231725 [https://www.ncbi.nlm.nih.gov/geo/query/acc.cgi?acc=GSE231725]. K562 and human brain transcriptomes, and various epigenomes from K562 and human/mouse brain curated from public databases and being re-analyzed in this study have been deposited in the GEO database under accession code GSE232215 [https://www.ncbi.nlm.nih.gov/geo/query/acc.cgi?acc=GSE232215]. The dbGAP data for the human CHEX samples is available as phs002120_v1 (https://www.ncbi.nlm.nih.gov/projects/gap/cgi-bin/study.cgi?study_id=phs002120.v1.p1 and https://ftp.ncbi.nlm.nih.gov/dbgap/studies/phs002120/phs002120.v1.p1/).
The sample sheet and the results of main analyses are available in Supplementary Data. Source data are provided with this paper.

Research involving human participants, their data, or biological material

Policy information about studies with human participants or human data. See also policy information about sex, gender (identity/presentation), and sexual orientation and race, ethnicity and racism.

Reporting on sex and gender	Human neurosurgical resections were used to generate primary cell cultures. The samples were deidentified prior to transport to the lab.
Reporting on race, ethnicity, or other socially relevant groupings	NA
Population characteristics	NA - Human cells came from human brain tissue that was resected at surgery. The samples were de-identified.
Recruitment	Patients were recruited prior to neurosurgery and asked if they would consent to use of their resected tissue in fundamental laboratory research.
Ethics oversight	University of Pennsylvania IRB approval #16223

Note that full information on the approval of the study protocol must also be provided in the manuscript.

Field-specific reporting

Please select the one below that is the best fit for your research. If you are not sure, read the appropriate sections before making your selection.

☒ Life sciences ☐ Behavioural & social sciences ☐ Ecological, evolutionary & environmental sciences

For a reference copy of the document with all sections, see nature.com/documents/nr-reporting-summary-flat.pdf

Life sciences study design

All studies must disclose on these points even when the disclosure is negative.

Sample size	As a pilot study no sample size estimation is done. Nevertheless, most of the analyses in this study are aggregates of available samples per biological/treatment group and main conclusions are drawn without group-wise comparison.
Data exclusions	All data are included in the paper and GEO deposit. Non-control samples with < 50 primed genes were called as outliers and excluded from downstream analyses.
Replication	For CHEX-seq, we set >=50 nonzero genes as the threshold to filter for good-quality samples. As a result, 17 mouse neuron culture samples were discarded. After filtering, we have at least 20 replicates for all biological groups except human astrocyte culture (n=7) and human neuron culture (n=12), and we have at least 3 replicates per time point for the TPA treated K562 group. For the gDNAzyme activity assays, each oligo or genomic DNA was tested a minimum of 3 times with each experiment being successful. Therefore, both CHEX-seq and gDNAzyme analysis were carried out based on a reasonable sample size of successful replicates.
Randomization	Neurons and astrocytes were randomly selected for analysis.

Blinding	As neurons and astrocytes were randomly selected for analysis there was no need for blinding.

Reporting for specific materials, systems and methods

We require information from authors about some types of materials, experimental systems and methods used in many studies. Here, indicate whether each material, system or method listed is relevant to your study. If you are not sure if a list item applies to your research, read the appropriate section before selecting a response.

Materials & experimental systems

n/a	Involved in the study
☐	☒ Antibodies
☐	☒ Eukaryotic cell lines
☒	☐ Palaeontology and archaeology
☐	☒ Animals and other organisms
☒	☐ Clinical data
☒	☐ Dual use research of concern
☒	☐ Plants

Methods

n/a	Involved in the study
☒	☐ ChIP-seq
☒	☐ Flow cytometry
☒	☐ MRI-based neuroimaging

Antibodies

Antibodies used	chicken Map 2 antibody, Abcam, Ab5392; Goat anti-chicken secondary antibody, Abcam, AB 150169
Validation	These antibodies have been used in 100's of manuscripts and have been verified by others with Western blotting and immunocytochemistry. Websites to companies validation - https://www.abcam.com/products/primary-antibodies/map2-antibody-ab5392.html and https://www.abcam.com/products/secondary-antibodies/goat-chicken-igy-hl-alexa-fluor-488-ab150169.html.

Eukaryotic cell lines

Policy information about cell lines and Sex and Gender in Research

Cell line source(s)	K562 cells were obtained from ATCC. Catalogue identifier - CCL-243
Authentication	cell lines were not authenticated in our lab, but it is the only human cell line used in the lab.
Mycoplasma contamination	cells lines were not tested for mycoplasma contamination
Commonly misidentified lines (See ICLAC register)	No commonly misidentified cell lines were used in these studies.

Animals and other research organisms

Policy information about studies involving animals; ARRIVE guidelines recommended for reporting animal research, and Sex and Gender in Research

Laboratory animals	C57Bl/6 mice - embryonic mouse brain cells were cultured for 2weeks after which they were used for CHEX analysis.
Wild animals	No wild anaimals were used in these studies.
Reporting on sex	The mouse primary cell cultures, were mixed sex cultures as all pups in a litter were used for brain and cell dissection, with no selection based upon sex.
Field-collected samples	No field-collected samples were used in these studies.
Ethics oversight	University of Pennsylvania IACUC oversight and approval.

Note that full information on the approval of the study protocol must also be provided in the manuscript.
